# Single beat 3D echocardiography for the assessment of right ventricular dimension and function after endurance exercise: Intraindividual comparison with magnetic resonance imaging

**DOI:** 10.1186/1476-7120-10-6

**Published:** 2012-02-22

**Authors:** Sebastian Schattke, Moritz Wagner, Robert Hättasch, Sabrina Schroeckh, Tahir Durmus, Ingolf Schimke, Wasiem Sanad, Sebastian Spethmann, Jürgen Scharhag, Alexander Huppertz, Gert Baumann, Adrian C Borges, Fabian Knebel

**Affiliations:** 1 Charité - Universitätsmedizin Berlin, Medizinische Klinik mit Schwerpunkt Kardiologie und Angiologie, Charité Campus Mitte, Charitéplatz 1, 10117 Berlin, Germany; 2 Institut für Radiologie, Charité Campus Mitte, Berlin, Germany; 3 Klinik für Dermatologie, Venerologie und Allergologie, Charité Campus Mitte, Berlin, Germany; 4 Innere Medizin III, Universitätsklinikum Heidelberg, Heidelberg, Germany; 5 Imaging Science Institute, Charité Berlin-Siemens, Berlin, Germany; 6 Klinik für Innere Medizin I, Kardiologie und Diabetologie, HELIOS Klinikum Emil von Behring, Berlin, Germany

**Keywords:** Single beat 3D echocardiography, Magnetic resonance imaging, Endurance exercise, Right ventricle

## Abstract

**Background:**

Our study compares new single beat 3D echocardiography (sb3DE) to cardiovascular magnetic resonance imaging (CMR) for the measurement of right ventricular (RV) dimension and function immediately after a 30 km run. This is to validate sb3DE against the "gold standard" CMR and to bring new insights into acute changes of RV dimension and function after endurance exercise.

**Methods:**

21 non-elite male marathon runners were examined by sb3DE (Siemens ACUSON SC2000, matrix transducer 4Z1c, volume rates 10-29/s), CMR (Siemens Magnetom Avanto, 1,5 Tesla) and blood tests before and immediately after each athlete ran 30 km. The runners were not allowed to rehydrate after the race. The order of sb3DE and CMR examination was randomized.

**Results:**

Sb3DE for the acquisition of RV dimension and function was feasible in all subjects. The decrease in mean body weight and the significant increase in hematocrit indicated dehydration. RV dimensions measured by CMR were consistently larger than measured by sb3DE.

Neither sb3DE nor CMR showed a significant difference in the RV ejection fraction before and after exercise. CMR demonstrated a significant decrease in RV dimensions. Measured by sb3DE, this decrease of RV volumes was not significant.

**Conclusion:**

First, both methods agree well in the acquisition of systolic RV function. The dimensions of the RV measured by CMR are larger than measured by sb3DE. After exercise, the RV volumes decrease significantly when measured by CMR compared to baseline.

Second, endurance exercise seems not to induce acute RV dysfunction in athletes without rehydration.

## Background

Extreme exercise might lead to right ventricular (RV) dysfunction and an elevated risk of arrhythmias in some athletes [1-7]. To acquire RV data cardiovascular magnetic resonance imaging (CMR) and echocardiography have been proven valuable tools.

Acquisition of RV echocardiographic data has been conventionally proven difficult due to its anterior position in the chest, a complex geometry and morphology with prominent trabeculations. Over the years several parameters have been developed to determine RV function by 2D echocardiography, e.g. Tricuspid Annular Plane Systolic Excursion (TAPSE), Tei-Index, systolic pulmonary arterial pressure (sPAP), strain and speckle tracking or even simple "eyeballing". It is, however, highly dependent on a standardized acquisition since a slight drift in angle of view results in a significant change of measurements [8]. Therefore the modality of choice for evaluating right ventricular chamber size and function is still considered to be cardiovascular magnetic resonance imaging. Recent advances in transducer and computing technology make single beat 3D echocardiography (sb3DE) possible. It might be an alternative that is less cost intensive than CMR and can be used on patients with pacemaker or an implantable cardioverter-defibrillator (ICD). Since it is single beat (shorter acquisition time) it is also applicable in patients with arrhythmias or breathing disorders. It is however very dependent on good image quality and a fairly wide ultrasound sector angle to include the entire right ventricle. Previous studies have shown that even though volumes acquired with sb3DE are generally smaller than acquired with CMR there is a fair correlation in the measured ejection fraction [9].

Several studies have already assessed changes of RV function and dimension after endurance exercise. Most of these studies used a one-time event like an official marathon race or even an ultra-endurance competition to acquire data. In such a setting CMR measurements are delayed due to logistical limitations. In most studies, CMR was performed within one or two days post-race and after rehydration.

The goals of this study were

1. to validate the new method of sb3DE of the RV against CMR and

2. to examine the immediate effect of endurance exercise on RV function.

In this study we examined amateur athletes immediately after a run of 30 km with CMR and sb3DE, in a randomized order and compared the results. No more than two runners ran at a time to ensure immediate measurement after exercise.

## Methods

### Subjects

Over a hundred male non-elite runners resident in the Berlin-area were asked to take part in this study. Inclusion criteria were a regular training routine and at least one finished marathon race in the past. Exclusion criteria were signs and symptoms of coronary heart disease, chronic cardiovascular disorders (atrial fibrillation, pacemaker, bypass surgery, prosthetic valves, congenital heart disease), allergy to gadolinium chelates or a known kidney disease.

The number of participants was limited in advance. The first 22 athletes were screened and provided baseline characteristics and medical and training history. Written informed consent was obtained from each participant. The institutional ethics committee approved the study protocol. The study complied with the Declaration of Helsinki.

### Study design

Each runner underwent baseline echocardiography, CMR and gave a blood sample one to three weeks prior to the 30 km run. The runners were asked to refrain from their regular training for four days before baseline testing to avoid elevated biomarkers or altered ventricular function.

There were neither pace nor fluid restrictions during the run. However, rehydration was prohibited after the race until completion of echocardiography and CMR measurements.

After the run echocardiography and CMR were performed in a randomized order (CMR before echocardiography in eleven cases) and a blood sample was taken.

### Echocardiography

Standard transthoracic echocardiography was performed by an experienced cardiologist according to the guidelines of the American Society of Echocardiography [10] on a Siemens ACUSON SC2000 echocardiography system (Siemens AG, Healthcare Sector, Erlangen, Germany, 4V1c 1,25-4,5 MHz transducer).

Sb3DE was performed using the same echocardiography system (4Z1c Instantaneous Full Volume transducer, 1,5-3,5 MHz). The runners lay in left decubitus position. A single beat scan was acquired from an apical 4-chamber transducer position in harmonic mode if needed for better border definition or fundamental mode during a single end-expiratory breath-hold. Depth and angle of the ultrasound sector were set to only visualize the right ventricle. Before each acquisition, images were optimized for endocardial border detection. At least two data sets were acquired per athlete to ensure optimal data quality. The volume rates were 10 to 29 frames per second. The data sets were stored digitally and were exported to a terminal workstation for further analysis.

**Figure 1:**
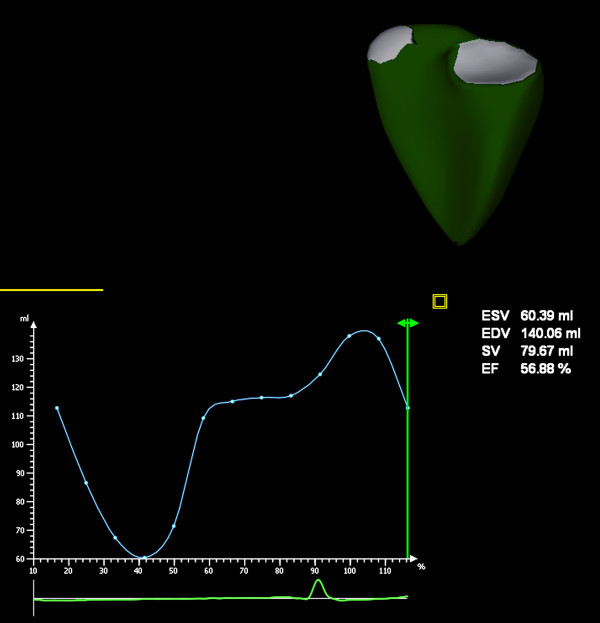
**Dynamic 3D model of the right ventricle**. The result of sb3DE analysis is displayed in a moving 3D model of the right ventricle. It provides a quick overview of anatomical conditions (e.g. dilatation), a curve showing right ventricular volume over the heart cycle and data in numbers

Data analysis was performed on a SC2000 workplace 1.5 (Siemens AG, Erlangen, Germany) running the Right Ventricular Analysis Application. This application requires manual tracing of the endocardial borders on a short-axis, apical 4-chamber and coronal view in end-systole and end-diastole. The detection of the ventricular surface throughout the cardiac cycle is then automated and represented in a dynamic 3D model (Figure 1).

### CMR

**Figure 2:**
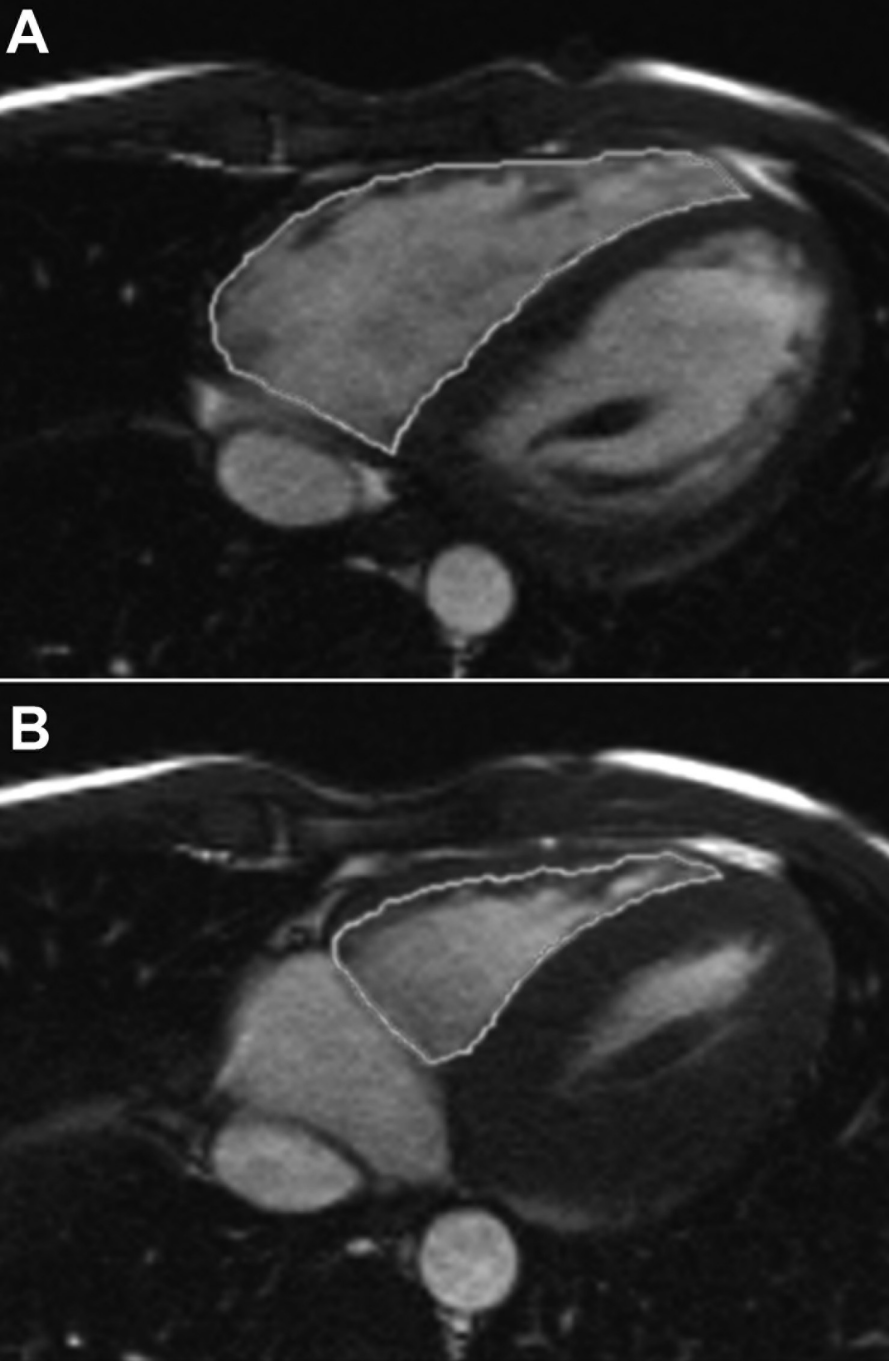
**Manual tracking of right ventricular endocardial borders in CMR**. Shown are two slices in the same height at the same examination. Endocardial borders in end-diastole (**A**) and end-systole (**B**) are traced. These discs are then calculated into a three-dimensional model of the ventricle

Cardiac magnetic resonance imaging was performed on a 1,5 Tesla MR system (Siemens Magnetom Avanto, Erlangen, Germany). Right ventricular volumes and function were evaluated on axial cine steady state free precession (SSFP) images (5 mm slice thickness)[11]. Calculation of RV end-diastolic volume (EDV), end-systolic volume (ESV), stroke volume (SV), and ejection fraction (EF) was performed using dedicated software (ARGUS, Siemens Medical Solutions, Erlangen, Germany) via manual tracking of RV endocardial borders in different slices and calculation of RV volumes using the Simpson method (Figure 2). RV trabeculations were included in the calculation of the ventricular volume [12].

The body surface area for the determination of cardiac index (CI) was calculated separately at the two time points (before and after the run) [13].

### Hematology

For hematologic parameters, EDTA plasma was collected and measured immediately.

### Statistical analysis

Results are expressed as mean value ± standard deviation, number (percentage) or coefficient (interval -1, +1). The Wilcoxon signed-rank test was used to compare repeated or related measurements (e.g. CMR RV EF baseline and post-run or CMR RV EF baseline and sb3DE RV EF baseline). Bland-Altman plots and the intraclass correlation coefficient (ICC) (two-way, mixed, average measure, according to Shrout [14]) were either used to describe the agreement of the two imaging modalities or for the assessment of conformity among observers. The limits of agreement for the Bland-Altman plot were specified as average difference ± two standard deviations of the difference [15].

For the calculation of intraobserver variability CMR and sb3DE volume analysis were repeated for four runners after three months. Three members of our group performing analysis of two data sets calculated echocardiography interobserver variability. They were blinded to each other's results.

## Results

22 athletes were screened. One runner showing signs of prior myocardial infarction (subendocardial delayed enhancement) in baseline-CMR was excluded from the study before the 30 km run. He was encouraged to undergo further cardiac diagnostics.

21 athletes ran in May and June 2010. All finished the 30 km run without adverse events. The average running time was 169,4 ± 17,4 minutes. The runners mean body weight decreased from 75,5 kg ± 7,9 to 73,2 kg ± 8,0. Table 1 shows the runners' baseline characteristics.

**Table 1:** Baseline characteristics

n	21
Age, y	45,7 ± 14,9

Body mass index, kg/m^2^	23,2 ± 1,90

Long-distance running experience, y	17,7 ± 10,7

Previous marathons, n	37,57 ± 66,1

Weekly average training, km	59,9 ± 24,3

Running time 30 km, min	169,4 ± 17,4

All post-run measurements were completed in less than 120 Minutes in all runners.

All subjects had sufficient image quality to perform sb3DE RV analysis. CMR showed no significant difference in the ejection fraction before and after the run, while RVEDV and RVESV and RVSV decreased significantly. (Table 2).

**Table 2:** Standard 2D Echocardiography, CMR and sb3DE data before and after exercise

n = 21	Baseline	post-run	p (baseline vs. post-run)
Heart rate, beats/min	57,6 ± 7,0	76,4 ± 13,8	**< 0,001**

Body weight, kg	75,5 ± 7,9	73,2 ± 8,0	**< 0,001**

**Standard-TTE**			

RVOT1, mm	25,3 ± 5,2	26,1 ± 6,0	0,826

RVOT2, mm	29,7 ± 3,4	27,8 ± 7,1	0,140

basal RV diameter, mm	38,2 ± 6,3	35,7 ± 6,0	0,262

mid RV SAX diameter, mm	33,1 ± 6,0	27,4 ± 14,0	0,424

RV long axis diameter, mm	82,6 ± 6,8	77,2 ± 12,6	0,221

RA diastolic area, cm^2^	11,0 ± 2,7	11,2 ± 3,9	0,484

RA systolic area, cm^2^	19,3 ± 3,2	17,4 ± 3,2	**0,030**

RV diastolic area, cm^2^	25,3 ± 3,6	22,5 ± 3,3	0,249

RV systolic area, cm^2^	13,2 ± 2,5	11,5 ± 2,6	0,116

FAC, %	48,1 ± 4,8	49,1 ± 7,1	0,463

PVAT, ms	178,7 ± 25,8	135,0 ± 35,2	**0,002**

PVET, ms	385,8 ± 51,7	321,0 ± 58,8	**0,002**

TAPSE, mm	29,6 ± 3,0	29,4 ± 5,6	0,270

RV E', cm/s	16,1 ± 3,3	15,3 ± 5,1	0,715

RV A', cm/s	16,4 ± 5,4	14,0 ± 8,7	0,465

RV S', cm/s	15,3 ± 3,1	12,8 ± 6,4	0,893

**CMR**			

RVEF, %	55,7 ± 6,4	54,0 ± 8,3	0,095

RVEDV, ml	216,0 ± 31,2	190,5 ± 30,9	**0,001**

RVESV, ml	96,3 ± 23,6	86,9 ± 19,0	**0,009**

RVSV, ml	119,6 ± 17,1	103,6 ± 22,5	**0,001**

**sb3DE**			

RVEF, %	53,0 ± 3,3	51,8 ± 4,0	0,230

RVEDV, ml	156,1 ± 44,4	144,6 ± 36,4	0,149

RVESV, ml	73,0 ± 19,8	70,4 ± 20,2	0,357

RVSV, ml	83,1 ± 25,4	74,2 ± 18,3	0,140

**cardiac index**			

CMR, l/min/m^2^	3,52 ± 0,4	4,13 ± 1,1	**0,007**

sb3DE, l/min/m^2^	2,46 ± 0,8	2,94 ± 0,9	**0,014**

Sb3DE as well showed no significant difference in the ejection fraction before and after the race. However, although the mean values were lower, sb3DE did not show a significant reduction of RVEDV, RVESV or RVSV.

Both methods, CMR and sb3DE, showed a significant increase of the CI.

Inter- and intra-observer variabilities and intraclass correlation coefficients are shown in Table 3.

**Table 3:** Intra- and inter-observer variabilities for CMR and sb3DE and intraclass correlation for sb3DE

		EF	EDV	ESV	SV
sb3DE	IntraOV, %	3,58	6,87	10,26	6,63
	InterOV, %	5,72	8,75	8,63	10,63
CMR	IntraOV, %	3,79	7,49	3,58	5,02

ICC (two way, mixed, consistency)					
sb3DE	IntraOC	0,51	0,87	0,77	0,90
	InterOC	0,61	0,96	0,99	0,89
CMR	IntraOC	0,96	0,98	1,00	0,95

Standard 2D echocardiography showed neither a significant change in fractional area change (FAC) nor in tricuspid annular plane systolic excursion (TAPSE). It did, however, show a significant decrease of pulmonary valve acceleration time (PVAT) and pulmonary valve ejection time (PVET).

**Figure 3:**
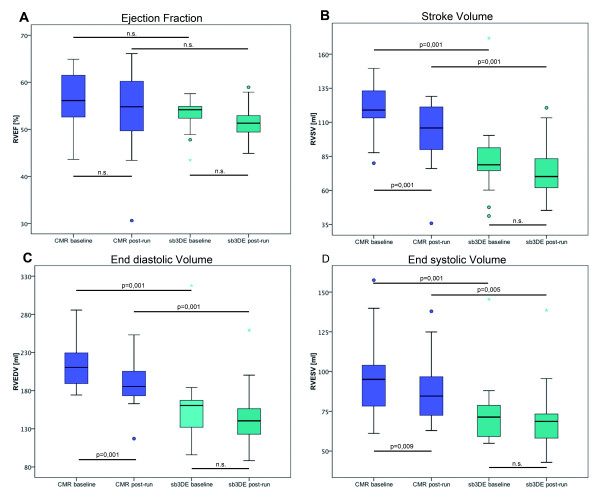
**Boxplots of RV function and dimensions measured by CMR and sb3DE**. RV ejection fraction (**A**), stroke volume (**B**), end-diastolic volume (**C**) and end-systolic volume (**D**) measured by CMR and sb3DE pre and post exercise. CMR shows larger volumes while the ejection fraction does not differ significantly

**Figure 4:**
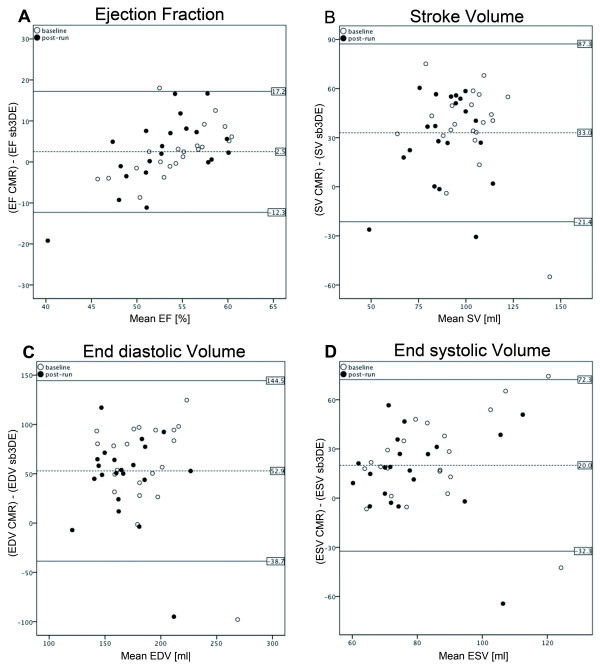
**Bland-Altman Plots for RV EF and dimensions comparing CMR and sb3DE**. Results of a Bland-Altman analysis of CMR versus sb3DE, showing the extent of agreement in RV ejection fraction (**A**), stroke volume (**B**), end-diastolic (**C**) and end-systolic volume (**D**). In a Bland-Altman analysis the mean values of the two modalities are assigned to the x-axis and the difference between the two modalities to the y-axis. The upper and lower line represents two times the standard deviation

The volumes measured by sb3DE were significantly smaller than those measured by CMR (Table 4). However, there was no significant difference between the EF determined by the two methods (Figure 3). Figure 4 shows Bland-Altman plots comparing RV ejection fraction and dimensions measured by both methods.

**Table 4:** Pooled pre- and post-exercise data of CMR and sb3DE

Pooled data, n = 42	CMR	sb3DE	p (CMR vs. sb3DE)
RVEF, %	54,9 ± 7,3	52,4 ± 3,67	**0,022**
RVEDV, ml	203,2 ± 33,3	150,3 ± 40,6	**< 0,001**
RVESV, ml	91,6 ± 22,7	71,7 ± 19,8	**< 0,001**
RVSV, ml	111,6 ± 21,3	78,6 ± 22,3	**< 0,001**

Cellular blood components (erythrocytes, leukocytes, thrombocytes, hematocrit) as well as sodium and potassium showed a relative increase.

## Discussion

This study is the first head-to-head comparison of sb3DE and CMR in the analysis of RV dimension and function immediately after endurance exercise in well-trained athletes. We found no signs of RV dysfunction. However, there was a decrease in RV volumes. This decrease was significant in CMR, in sb3DE the mean values tended to be lower after the run but a high heterogeneity resulted in no significance.

Sb3DE was feasible in all subjects. Acquisition and RV analysis is possible within minutes. Sb3DE can be performed in tachycardic subjects after exercise. Data analysis in sb3DE is dependent on good image quality. While detection of the endocardial border was easily possible in the apical 4-chamber view it often was a challenge in short-axis and coronal views. To facilitate contour delineation, the software gives reference points based on the users previous input in former views. This, although being a helpful feature, makes measurement prone for sequence errors. Due to the RV anterior position in the chest, the RV anterior wall and outflow tract in the near field remains problematic in some subjects despite good image quality. This is a reason for the relatively high intra- and inter-observer-variability, which might be more pronounced in patients with RV disease or poor acoustic windows. An advantage of single beat 3D echocardiography is the lack of stitching artifacts and prolonged breath-hold, which will lead to a better acceptance of the method in routine echocardiography. Previous studies have used stitched volumes. The normal values for this method were published recently [8,16]. Compared to these normal values, our subjects had larger ventricles; this is most likely due to the fact that we have examined well trained athletes [17]. The RVEF in our subjects is lower than the published normal values [16]. Still, it is in normal range in all subjects before and after endurance exercise. The intra- and inter-observer-variability in our study is comparable with published data [16,18].

Although CMR is considered as the gold standard for RV volumetry and functional analysis [19] a clear identification of the tricuspid or pulmonary valve or the endocardial border is not always possible. Also, border delineation is user-dependent (e.g. how much trabeculation is included). In addition, relevant restrictions and contraindications have to be considered. Its use is limited in children, claustrophobic patients and patients with pacemakers or similar implants. Furthermore, it is relatively expensive and not easily available. Image acquisition requires a sufficient patient compliance.

Both sb3DE and CMR have relatively low volume/frame rates. Therefore, the definition of end-diastole and end-systole can be missed. This is especially difficult if the patient has a high heart rate (in our study the highest post-run heart rate was 111/minute).

RV dimensions and function by sb3DE were in fair correlation to CMR. As previously described, sb3DE defines lower volumes than CMR [9,20,21]. This is partly due to differences in data analysis since in sb3DE the endocardial borders are traced and most of the trabeculations are excluded while generally in CMR most of the trabeculations were included into the calculation of the cavity volume. In CMR analysis this is clinical standard because it leads to a faster and more reliable analysis [12,22].

The discrepancy between volumetry in 3D echocardiography and CMR is more pronounced in larger ventricles [22,23]. RV 3D echocardiography was recently validated against CMR in patients with congenital heart disease [9,20]. Only 50-80% of the patients had a sufficient acoustic window for 3D echocardiography. In our study with ideal healthy subjects 100% could be analyzed. The RV analysis algorithm is designed for normal shaped RV and not congenital RV disease. Additionally, several data sets were acquired per athlete to ensure optimal data quality.

In sports cardiology, the issue of RV dysfunction after endurance exercise is not solved. Most CMR studies had a long delay of data acquisition after the race or did not mention the time point of echocardiography and CMR [3,6,24]. Neither CMR, nor sb3DE or standard transthoracic echocardiography showed RV systolic dysfunction in dehydrated subjects in our study. The changes of pulmonary valve acceleration and ejection time are within normal limits and probably due to the change in heart rate. Thus they do not indicate the presence of post-exercise pulmonary hypertension.

All other previously published studies that have investigated RV function after exercise have measured after rehydration and showed RV dysfunction [3,6,24].

Our laboratory results clearly underline the presence of relevant dehydration.

Our study is limited by the fact that we have not performed additional echocardiography and CMR after rehydration. It therefore remains unclear if rehydration through an increase in volume load leads to a change in RV function.

## Conclusions

Both CMR and sb3DE are feasible in healthy athletes with high and low heart rates.

The two modalities showed a good correlation of RV function. Sb3DE obtained, however, smaller RV volumes compared to CMR. In conclusion, it is a sufficient alternative whenever it comes to image acquisition that needs to be done fast, with less expenditure or with patients that have contraindications for CMR.

In contrast to previous data, we could not detect RV dysfunction after exercise in either method. We assume that rehydration and volume load were important factors, which needs further investigation.

## Abbreviations

CI: Cardiac index; CMR: Cardiovascular magnetic resonance imaging: EDTA: Ethylenediaminetetraacetic acid; EDV: End-diastolic volume; EF: Ejection fraction; ESV: End-systolic volume; FAC: Fractional area change; Hs-cTnT: High sensitivity cardiac troponin T; ICC: Intraclass correlation coefficient; ICD: Implantable cardioverter-defibrillator; IL-6: Interleukin 6; IOV: Interobserver-variability; NT-proBNP: N-terminal prohormone of brain natriuretic peptide; PVAT: Pulmonary valve acceleration time; PVET: Pulmonary valve ejection time; RV: Right ventricle: right ventricular; RVOT: Right ventricular outflow tract; SAX: Short axis; Sb3DE: Single beat three dimensional echocardiography; SCD: Sudden cardiac death; sPAP: systolic pulmonary arterial pressure; SV: Stroke volume; TAPSE: Tricuspid annular plane systolic excursion.

## Competing interests

The authors declare that they have no competing interests.

## Authors' contributions

SS, Conception and design of the study, analysis and interpretation of echocardiographic data, revision of the manuscript. MW, Conception and design of the study, analysis and interpretation of CMR data, revision of the manuscript. RH, Conception and design of the study, analysis and interpretation of CMR and echo data, drafting and revision of the manuscript, SS, Conception and design of the study. TD, Conception and design of the study, analysis and interpretation of CMR data, revision of the manuscript. IS, Analysis and interpretation of hematologic data. WS, Conception and design of the study, analysis and interpretation of echocardiographic data, revision of the manuscript. SS Revision of the manuscript. JS, Revision of the manuscript. AH, Conception and design of the study. GM, Conception and design of the study. ACB, Conception and design of the study. FK, Conception and design of the study, analysis and interpretation of echocardiographic data, drafting and revision of the manuscript. All authors read and approved the final Manuscript.
